# An Underwater Polarization Imaging Technique Based on the Construction and Decomposition of the Low-Rank and Sparse Matrix in Stokes Space for Polarization State Imaging

**DOI:** 10.3390/s25030704

**Published:** 2025-01-24

**Authors:** Pengfeng Liu, Yuxiang Zhai, Hongjin Zhu, Zijian Ye, Qinyu He, Zhilie Tang, Peijun Tang

**Affiliations:** 1 School of Physics, South China Normal University, Guangzhou 510006, China; 2021022395@m.scnu.edu.cn (P.L.); yxiang0225@163.com (Y.Z.); 2021022355@m.scnu.edu.cn (H.Z.); 13080924459@163.com (Z.Y.); hqy142857@163.com (Q.H.); 2 Guangzhou Institute of Science and Technology, Guangzhou 510540, China; 3 School of Optoelectronic Science and Engineering, South China Normal University, Guangzhou 510006, China

**Keywords:** polarization imaging, Stokes parameters channels, low-rank and sparse matrix, reconstruction

## Abstract

Traditional underwater polarization imaging methods can only provide clear degree of polarization (DOP) and intensity images of the object but cannot provide images of the polarization state of the object. This paper proposes a method to extract clear object information from turbid water in all four Stokes parameter (I, Q, U, and V) channels by using the full Stokes camera, enabling clear polarization state image reconstruction. The method utilizes multiple images from different angles to construct a low-rank and sparse matrix. Then, by decomposing this matrix into sparse and low-rank components, clear Q, U, and V images (i.e., the full polarization state) can be obtained. Unlike traditional methods that assume the circularly polarized component (V component) to be zero, this method retains V channel information, allowing for circular polarization component measurement. The study successfully reconstructed clear underwater images of samples with inhomogeneous DOP distribution and obtained the clear polarization states of polarizers and fish in the turbid water. The results show that the proposed method can visualize and analyze the object’s polarization state quantitatively with high accuracy in turbid water for the first time, potentially extending the applicability of polarization underwater imaging in ocean exploration.

## 1. Introduction

The constraints on human research and exploration of marine badlands are far more complex than those on land. Because of its high information content, high imaging resolution, and fast frame speed, underwater optical imaging technology has a wide range of applications in the fields of marine resource exploration, monitoring of water quality, and localization of underwater targets [1,2,3]. However, underwater optical imaging technology is constrained by complex environmental factors such as absorption and scattering in water bodies, especially in turbid water bodies, where the strong scattering effect leads to difficulty in improving the quality of underwater imaging [4]. Many underwater clear imaging techniques have been proposed to solve these problems, such as image enhancement techniques [5,6], structured light imaging [7,8], simultaneous scanning imaging techniques [9], polarization imaging techniques, etc. [10,11]. Image enhancement techniques can mitigate the image distortion that is introduced by the scattering of ambient light in underwater environments and improve the clarity and contrast of objects. However, the image enhancement techniques do not consider the fundamental reasons for image degradation, due to which the processed images are unable to represent the original information accurately and suffer noise amplification, especially in higher concentration environments. Imaging techniques based on optical imaging models, such as structured light imaging and synchrotron scanning imaging, typically require relatively complex hardware devices and highly customized systems, further increasing the complexity of the implementation and making it subject to numerous limitations in terms of cost and imaging speed [12]. Polarization imaging techniques are widely used due to the advantages of simple equipment manufacturing, low cost, and low environmental constraints on image quality.

The polarization imaging technique utilized the difference of the polarization information to separate the ambient light and the object information. The underwater passive polarization imaging technique proposed by Schechner et al. [13] achieves clear underwater images by exploiting the difference in the polarization characteristics between the ambient light and the object light in the underwater environment. However, this technique is based on the assumption that the degree of polarization (DOP) of the ambient light is constant and that of the sample is zero. Treibitz et al. [14] considered and computed the DOP of the object but still did not consider the inhomogeneous distribution of the polarization of the ambient light and the object. Previous studies [15,16] proposed a method that utilized low-rank sparse decomposition (LRSD) and decorrelation methods to estimate the distribution of the DOP and angle of polarization of the ambient light and the object. Then, the ambient light and the object can be separated by independent component analysis (ICA). The method proposed by this group can provide a clear DOP image of the target but cannot provide the full polarization state information (i.e., I, Q, U, and V) of the target and ambient light simultaneously, which is important and can show more polarization information of the target. Moreover, the models proposed by this group are all based on the assumption that the circularly polarized component (i.e., the V component) of the light is zero, which is not close to some practical situation, especially for the light scattered back from the highly birefringent object. The method proposed by this group requires relatively complicated computation, including LRSD, decorrelation, ICA, and so on, which is relatively time-consuming. Hence, there is a need to propose a facile method that can provide a clear full polarization state image of the object in the underwater environment to provide more polarization information about the object.

To extract the clear underwater images with full polarization state information (including the circular polarized component V), a facile underwater imaging method that can separate the object information from the ambient light in each of the four Stokes parameters channels (I, Q, U, and V), respectively, is proposed in this paper. Specifically, this method is implemented by capturing multiple images of the objects with different angles by moving a full Stokes camera, which can capture the four Stokes parameters simultaneously. Then, each Stokes parameters channel has multiple images from different imaging angles. For each Stokes parameter, by rearranging the images with different angles into a new low-rank and sparse matrix, the ambient light and the object can be separated as the low-rank part and the sparse part based on the LRSD method because of the correlation of the ambient light and the sparsity of the object in the multiple images. By separating the object information in each of the Stokes parameters channels, the full polarization state information of the object without the distortion introduced by the ambient light can be obtained with improved contrast and can be visualized in one figure by using the color-encoding method [17] in the color space. The experimental results show that the proposed method can image the sample with high (ruler) and low DOP (tape) simultaneously with improved contrast and signal-to-noise ratio (SNR) by considering the polarization distribution of the ambient light and the object in the 4 Stokes parameters channels, respectively. Most importantly, the clear polarization state images of the polarizers with different orientations are reconstructed efficiently. The polarization orientations of the light scattered back from different polarizers are obtained in the underwater environment with high accuracy.

## 2. Methods and System Setup

To demonstrate the necessity of the consideration of the distribution of DOP of the object, the main theory of Treibitz’s method is presented first. The intensity of light received by the camera can usually be regarded as an incoherent superposition of the background scattered light and the object’s reflected light. The active polarization underwater imaging model [14] uses polarized light active illumination, estimates the polarization of the background scattered light and the object’s reflected light as a constant, and performs a clear image of the object. As in Equation (1),

(1) $$\begin{array}{cc} S= & \frac{1}{P_{B}-P_{S}}\left[I_{min}\times \left(1+P_{B}\right)-I_{max}\times \left(1-P_{B}\right)\right];\\ B= & \frac{1}{P_{B}-P_{S}}\left[I_{max}\times \left(1-P_{S}\right)-I_{min}\times \left(1+P_{S}\right)\right]. \end{array}$$

In the active polarization underwater imaging model, *S* represents the target light, *B* represents the backscattered light, $I_{max}$ and $I_{min}$ represent the maximum and minimum light intensity images obtained from polarized detection, and $P_{S}$ and $P_{B}$ represent the polarization degrees of the target and backscattered light, respectively. Many studies are dedicated to optimizing two parameters: $P_{S}$ and $P_{B}$. Treating $P_{B}$ and $P_{S}$ as constants leads to the incomplete removal of backscattered light, and the inability to image the polarization distribution of the objects [18,19,20,21,22,23,24,25].

In this paper, we consider the distribution of the background scattered light and the object reflected light in the Stokes parameters channels [I Q U V]. The light intensity in each Stokes parameters channel can be regarded as an incoherent superposition of the intensity of the background scattered light, and the object reflected light because the underwater environment makes the coherence of the light relatively low, and the light source we used is an incoherent LED [26]. As in Equations (2)–(4),

(2) $$\left[\begin{array}{c} I_{targe}\\ Q_{targe}\\ U_{targe}\\ V_{targe} \end{array}\right]=\left[\begin{array}{c} I_{targe}\\ I_{targe}P_{targe}\cos2\varphi_{1}\cos2\chi_{1}\\ I_{targe}P_{targe}\sin2\varphi_{1}\cos2\chi_{1}\\ I_{targe}P_{targe}\sin2\chi_{1} \end{array}\right].$$

$\left[I_{target}\;Q_{target}\;U_{target}\;V_{target}\right]$ denotes the Stokes vector of the reflected light from the object, $P_{targe}$ is the polarization of the objects reflecting light, and $\varphi_{1}$ and $\chi_{1}$ are the phase angle and ellipticity angle of the objects reflecting light.

(3) $$\left[\begin{array}{c} I_{back}\\ Q_{back}\\ U_{back}\\ V_{back} \end{array}\right]=\left[\begin{array}{c} I_{back}\\ I_{back}P_{back}\cos2\varphi_{2}\cos2\chi_{2}\\ I_{back}P_{back}\sin2\varphi_{2}\cos2\chi_{2}\\ I_{back}P_{back}\sin2\chi_{2} \end{array}\right].$$

$\left[I_{back}\;Q_{back}\;U_{back}\;V_{back}\right]$ denotes the Stokes vector of ambient scattered light, $P_{back}$ is the polarization of the background scattered light, and $\varphi_{2}$ and $\chi_{2}$ are the phase angle and ellipticity angle of the background scattered light.

(4) $$\left[\begin{array}{c} I_{total}\\ Q_{total}\\ U_{total}\\ V_{total} \end{array}\right]=\left[\begin{array}{c} I_{total}\\ I_{total}P_{total}\cos2\varphi \cos2\chi \\ I_{total}P_{total}\sin2\varphi \cos2\chi \\ I_{total}P_{total}\sin2\chi \end{array}\right].$$

$\left[I_{total}\;Q_{total}\;U_{total}\;V_{total}\right]$ represents the Stokes vector received by the camera. From Equations (2)–(4), due to the underwater environment and the LED light source, the Stokes parameters received by the camera can be expressed as an incoherent superposition of the reflected light from the object and the Stokes vector of the ambient scattered light as shown in Equation (5).

(5) $$\left[\begin{array}{c} I_{total}\\ Q_{total}\\ U_{total}\\ V_{total} \end{array}\right]=\left[\begin{array}{c} I_{back}+I_{target}\\ Q_{back}+Q_{target}\\ U_{back}+U_{target}\\ V_{back}+V_{target} \end{array}\right].$$

By moving the camera multiple times while keeping the camera and the light source relatively stationary, multiple images can be obtained. Since the camera is a full Stokes camera, the four Stokes parameters can be captured simultaneously. Due to the Brownian motion of the scattering particles in the water, the particle concentration is approximately the same everywhere in the turbid liquid, so the background scattered light distribution in multiple images is highly correlated. We rearranged these multiple images into a row vector and thus constructed a new matrix space including multiple images, as shown in Equation (6). Because the distribution of the background scattered light in multiple images is highly correlated, the background scattered light has a low rank in the constructed matrix space. The target object of underwater imaging is generally far away and occupies a small region in the field of view (FOV), so the object has a sparse characteristic in the matrix space. To summarize, the constructed matrix space can be regarded as the sum of two matrices, a low-rank matrix composed of background scattered light and a sparse matrix composed of objects, as shown in Equation (6).

(6) $$\begin{array}{cc} D & =\left[\begin{array}{c} I_{total1}\\ I_{total2}\\ I_{total3}\\ I_{total4}\\ \cdots \cdots \end{array}\right]=\left[\begin{array}{c} I_{back1}+I_{target1}\\ I_{back2}+I_{target2}\\ I_{back3}+I_{target3}\\ I_{back4}+I_{target4}\\ \cdots \cdots \end{array}\right]\\ \; & =\left[\begin{array}{c} I_{back1}\\ I_{back2}\\ I_{back3}\\ I_{back4}\\ \cdots \cdots \end{array}\right]+\left[\begin{array}{c} I_{target1}\\ I_{target2}\\ I_{target3}\\ I_{target4}\\ \cdots \cdots \end{array}\right]=A+E \end{array}.$$

To extract the clear target information, we just need to carry out the low-rank and sparse decomposition of matrix D. The solution to this problem can be described as Equation (7).

(7) $$\begin{array}{ccc} \min_{A,E}\; & \; & rank\left(A\right)+{\lambda ∥E∥}_{0}\\ s.t. & \; & D=A+E \end{array},$$

where rank(A) and ${\lambda ∥E∥}_{0}$ are nonlinear and nonconvex and are very difficult to optimize; this problem is also known as principal component pursuit (PCP). ${∥∥}_{*}$ and ${∥∥}_{1}$ can be performed in order to solve the above problem. Since the kernel paradigm of a matrix is a convex envelope of the matrix rank and the ${∥∥}_{1}$ paradigm of a matrix is a convex envelope of ${∥∥}_{0}$, the problem can be relaxed into the following convex optimization problem:

(8) $$\begin{array}{ccc} \min_{A,E}\; & \; & {∥A∥}_{*}+\lambda {∥E∥}_{1}\\ s.t. & \; & D=A+E \end{array}.$$

For Equation (8), the generalized Lagrange multiplier algorithm, known as the alternating direction method of multiple (ADMM) [27,28,29], is used to solve it in this paper. As long as the singular values of the low-rank matrix are reasonably distributed and the nonzero elements of the sparse matrix are uniformly distributed, the convex optimization problem is able to separate the original low-rank matrix and the sparse matrix from the unknown arbitrary error with a probability close to one.

According to Equation (5), the Stokes vector received by the camera can be expressed as a linear superposition of the reflected light from the object and the scattered light from the environment. Then, the other three components can also be regarded as a superposition of low-rank and sparse matrices, as shown in Equations (9)–(11):

(9) $$\left[\begin{array}{c} Q_{total1}\\ Q_{total2}\\ Q_{total3}\\ Q_{total4}\\ \cdots \cdots \end{array}\right]=\left[\begin{array}{c} Q_{back1}\\ Q_{back2}\\ Q_{back3}\\ Q_{back4}\\ \cdots \cdots \end{array}\right]+\left[\begin{array}{c} Q_{target1}\\ Q_{target2}\\ Q_{target3}\\ Q_{target4}\\ \cdots \cdots \end{array}\right],$$

(10) $$\left[\begin{array}{c} U_{total1}\\ U_{total2}\\ U_{total3}\\ U_{total4}\\ \cdots \cdots \end{array}\right]=\left[\begin{array}{c} U_{back1}\\ U_{back2}\\ U_{back3}\\ U_{back4}\\ \cdots \cdots \end{array}\right]+\left[\begin{array}{c} U_{target1}\\ U_{target2}\\ U_{target3}\\ U_{target4}\\ \cdots \cdots \end{array}\right],$$

(11) $$\left[\begin{array}{c} V_{total1}\\ V_{total2}\\ V_{total3}\\ V_{total4}\\ \cdots \cdots \end{array}\right]=\left[\begin{array}{c} V_{back1}\\ V_{back3}\\ V_{back3}\\ V_{back4}\\ \cdots \cdots \end{array}\right]+\left[\begin{array}{c} V_{target1}\\ V_{target2}\\ V_{target3}\\ V_{target4}\\ \cdots \cdots \end{array}\right].$$

By applying ADMM to (9) and (11), the ambient light and the object light can be separated by the ADMM in each of the Stokes parameters channels [I, Q, U, V]. Then, the clear polarization state (i.e., Q, U, and V) image of the object can be obtained. By using red, green, and blue to encode the Q, U, and V images, respectively, as shown in Equation (12), the clear polarization state image of the object can be visualized in the color-space in one image:

(12) $$\begin{array}{c} R=\frac{1}{2}\left(\frac{Q_{target}}{I_{target}}+1\right),\\ G=\frac{1}{2}\left(\frac{U_{target}}{I_{target}}+1\right),\\ B=\frac{1}{2}\left(\frac{V_{target}}{I_{target}}+1\right). \end{array}$$

**Figure 1:**
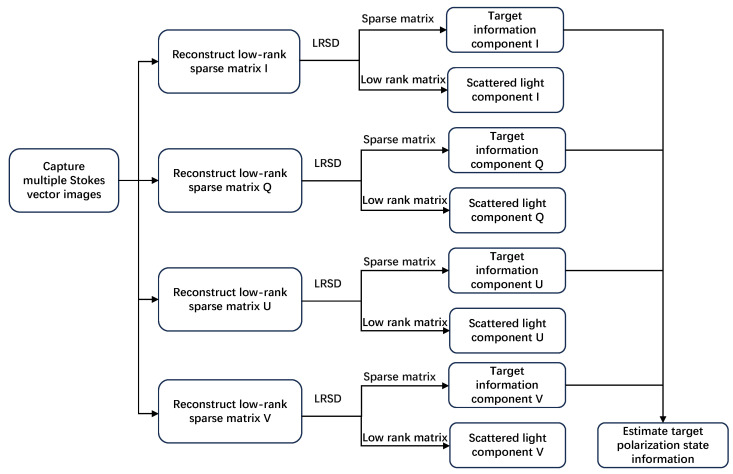
A flowchart of the underwater polarization state imaging algorithm.

The flowchart of the imaging algorithm is shown in Figure 1.

**Figure 2:**
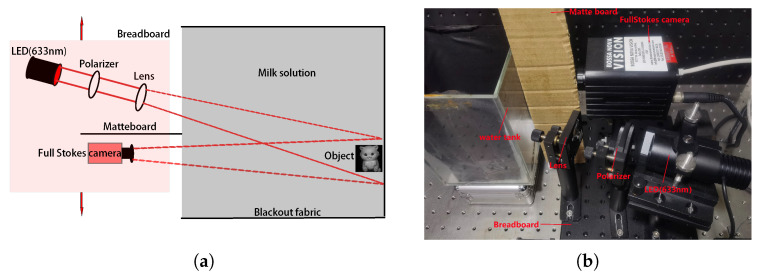
(**a**) Equipment diagram. (**b**) Physical diagram of the experimental device.

The diagram of the underwater polarization imaging system is shown in Figure 2a. A monochromatic LED (DH-PX30-R) with a wavelength of 633 nm was used as the light source. The camera was a full Stokes polarization camera (Bossa Nova vision). The physical diagram of the experimental device is shown in Figure 2b. The monochrome LED red light is sequentially passed through the diaphragm, polarizer, and lens. Different from the conventional cameras that capture the intensity of light, the Stokes polarization camera is able to quickly record the four Stokes parameters of light [I Q U V] simultaneously. By analyzing the four polarization components, the Stokes polarization camera can provide the complete polarization state of light. The camera was pre-calibrated for 633 nm monochromatic light. A board was put between the light source and the camera to prevent the container wall from reflecting light into the imaging system. The object to be imaged was placed in a mixture of water and milk, and the wall of the container was covered with black fabric with the purpose of reducing the experimental error caused by the reflection from the container wall. The glass container has a volume of 12cm×12cm×20cm, and different volumes of skimmed milk are added to simulate the absorption and scattering of light by suspended particles in a real underwater environment.

The mobile device imaging mode is shown in Figure 2b. In this study, the light source and the camera were fixed on a breadboard (YH-2030-13), with the angle between the light source and the polarized camera set to approximately 20°. Since the positions of the light source and the camera were fixed on the breadboard, their relative position and angle remained unchanged during the movement of the breadboard. As the distribution of backscattered light is primarily determined by the scattering characteristics of the light source, its distribution in the camera is almost unaffected by changes in the camera’s position. Therefore, regardless of the position of the light source-camera system underwater, the distribution of backscattered light can be considered consistent. This consistency ensures that the backscattered light in the image sequence can be modeled as a low-rank component while the target region, due to its sparsity, occupies only a small number of pixels or a localized area in each image. We moved the entire light source-camera system multiple times and captured images of the target from five different perspectives. During each capture, the relative position of the light source and the camera remained unchanged, with only the system’s position and angle relative to the target being adjusted. The image acquisition parameters included a camera exposure time of 40 ms and a lens focal length of 15 cm, ensuring clear and high-quality images. This setup not only guaranteed the reliability of the data but also provided rich multi-angle information for subsequent analysis.

## 3. Experimental Results

**Figure 3:**
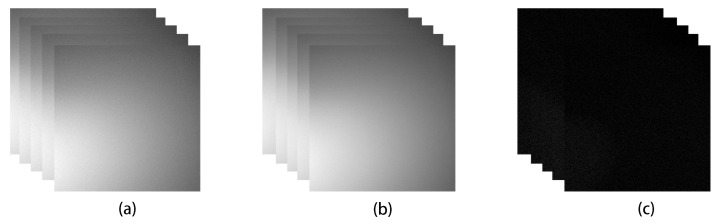
(**a**) A group of intensity images (*I*) of the ambient light. (**b**) Separated low-rank components obtained by the proposed method. (**c**) Separated sparse components obtained by the proposed method.

The rank of the matrix represents the maximum number of linearly independent rows or columns in the matrix. When the elements in the matrix are highly correlated, the rank of the matrix is low. To demonstrate that the background scattered light distribution in the multiple images with different angles is highly correlated, we performed imaging experiments in a turbid water body without objects. By moving the light source and camera and taking five shots, the group of the intensity images (i.e., *I*) was obtained, as shown in Figure 3.

By rearranging the group of intensity images into the new matrix (Equation (6)) and applying the ADMM to the matrix, the low-rank component and the sparse component of this matrix are shown in Figure 3b and Figure 3c, respectively. It can be seen that the background scattered light lies almost entirely in the low-rank part, and the sparse matrix is almost zero, demonstrating that the ambient light can be separated as the low-rank component by the proposed method.

**Figure 4:**
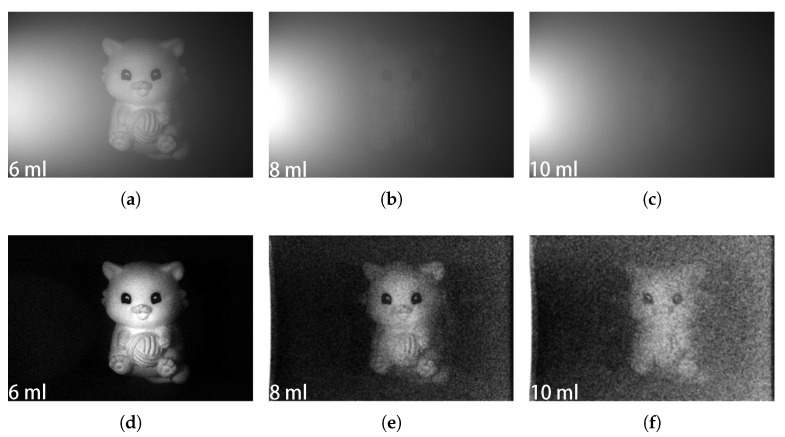
Polarization underwater imaging of the plastic doll (low DOP) in different concentrations of the milk solution. (**a**–**c**) are the original intensity images with the milk concentrations of 6, 8, and 10 mL/L, respectively. (**d**–**f**) are recovered intensity images of the plastic doll with the milk concentrations of 6, 8, and 10 mL/L, respectively, using the proposed LRSD-based method.

The plastic doll, which is a highly depolarized object in a milk solution with different concentrations of 6 mL/L, 8 mL/L, and 10 mL/L, was imaged by the proposed system. The raw intensity images corresponding to different concentrations are shown in Figure 4a–c. It can be seen that the ambient light scattered by the milk makes the objects more and more blurred as the concentration increases. By applying the proposed method to the matrix constructed by the group of intensity images, the ambient light can be removed, and the object is clearer and more visible, as shown in Figure 4d–f. In terms of shape preservation, the original shape of the plastic doll is maintained during the image reconstruction process, and the edges and overall structure are preserved without excessive smoothing or deformation. In terms of noise suppression, the method successfully mitigates the effect introduced by the underwater backscattered light, ensuring a clean visualization. Even in high-concentration environments, as shown in Figure 4e,f, the method is still able to effectively restore the doll’s image, maintain clarity, and accurately reproduce details and structures. This demonstrates the robustness, adaptability, and reliability of the algorithm for dealing with challenging scenes such as high-concentration environments. Moreover, these results demonstrate that the proposed method is suitable for the object with high depolarization.

**Figure 5:**
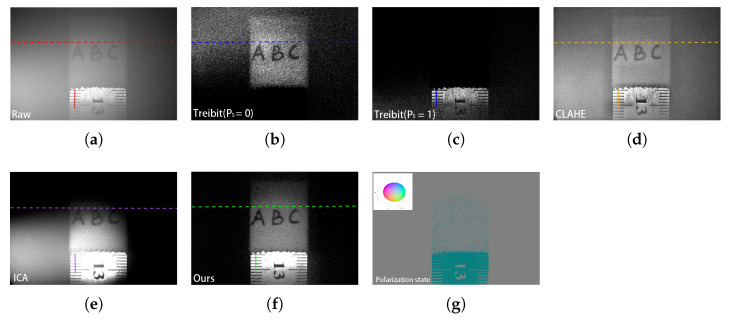
Polarization underwater imaging of the iron ruler (high DOP) and tape (low DOP) in a milk solution with the concentration of 6 mL/L. (**a**) Original underwater intensity image. (**b**) Image reconstructed by Treibitz’s method by setting the DOP of the sample as 0 ($P_{S}$ = 0). (**c**) Image reconstructed by Treibitz’s method by setting the DOP of the sample as 1 ($P_{S}$ = 1). (**d**) Image reconstructed by CLAHE’s method. (**e**) Image reconstructed by ICA’s method. (**f**) Intensity image reconstructed by the proposed method. (**g**) Color-encoded polarization state image reconstructed by the proposed method.

To demonstrate that the proposed method can image the objects with high DOP and low DOP simultaneously, an iron ruler (high DOP) wrapped with tape (low DOP) is imaged in the same FOV by the proposed method. The results of the iron ruler and the tape are shown in Figure 5. Figure 5a shows the raw intensity image of the ruler and tape, where the ruler and the tape are blurred by the ambient light. In the traditional Trebitz’s method (Equation (1)), the DOP of the object is considered as a constant. By considering the DOP of the object as 0, only the object with low DOP (i.e., the tape) can be visualized in Figure 5b. while by considering the DOP of the object as 1, only the ruler can be visualized in Figure 5c. The two objects with different DOPs cannot be visualized simultaneously in the whole FOV because this method neglects the distribution of the DOP of the sample. A pure contrast-enhancement technique, which is called the histogram equalization (CLAHE) method [30], is utilized to enhance the intensity of the image. Although this method can improve the image contrast to some extent, it does not effectively remove the ambient light (Figure 5d). Figure 5e is the image after ICA processing. For underwater images with low signal-to-noise ratio (SNR), noise dominates the principal components. The signals separated by ICA may be significantly affected, and noise may even be mistaken for independent components, leading to the inability to separate targets in low SNR areas. Figure 5f shows the result reconstructed by the proposed method in the intensity channel. It can be seen that not only the ambient light can be mitigated efficiently, but both the ruler (high DOP) and the tape (low DOP) can be visualized simultaneously with high contrast, demonstrating that the proposed method can image the objects with different DOP simultaneously. This is because the model proposed by this study considers the distribution of the DOP of the ambient light and the object. To obtain the full polarization state information of the object, the proposed method is applied to the other three Stokes parameters channels, respectively. Clear images in the Q, U, and V channels were obtained and were coded by red, green, and blue using Equation (12). Then, the polarization state information of the objects is shown in Figure 5g. It can be seen that the colors of the tape are grayish because the light scattered back from the tape is highly depolarized and, hence, is close to zero. However, the polarization state of the ruler (i.e., [−0.93 0.02 0]), which is close to that of the incident light as expected because the ruler is an amorphous sample with high DOP, can preserve the polarization state of the incident light. Each point at the Poincare sphere surface represents a unique polarization state. By using the color-encoded method (i.e., using the red, blue, and green to code the Q, U, and V values), the surface of the Poincare sphere can be color-encoded. Hence, we utilized the color-encoded Poincare sphere as the colormap of the polarization state. In all the following figures, the color represents a unique polarization state at the Poincare sphere surface.

**Figure 6:**
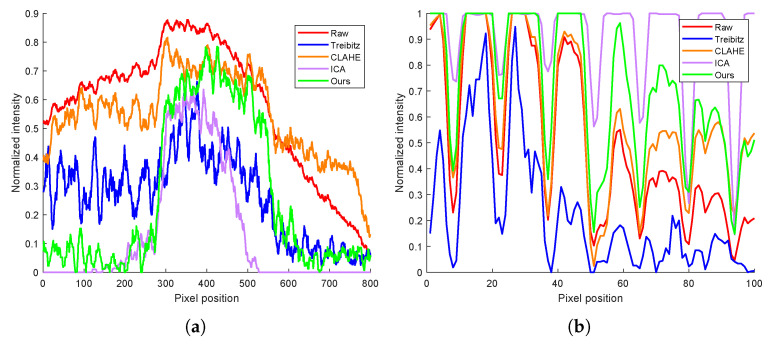
Curves of the grayscale values. (**a**) Gray value of the dotted line in Figure 5. (**b**) Gray value of the solid line in Figure 5.

Figure 6 shows the curves of the grayscale values of the position indicated by the dotted and solid lines in Figure 5. Green curves represent the grayscale values in the images reconstructed by the proposed method. It can be seen that compared with the curves provided by the raw underwater image and the images reconstructed by Trebitz’s method and CLAHE’s method, the proposed method can provide the best contrast. To demonstrate that the proposed method indeed can provide a better performance for underwater imaging, the peak signal-to-noise ratio (PSNR), the enhancement measure evaluation (EME), and the structural similarity index measure (SSIM) of the images reconstructed by different methods were computed, as shown in Table 1. It can be seen that our method improves the PSNR of the image from 8.05 to 17.46 by a factor of 2.17 compared with the raw image and the EME of the image from 6.09 to 20.43 by a factor of 3.35 compared with the raw image. Additionally, the SSIM for the raw image is 0.4346, while our method achieves an SSIM of 0.7156. All of these improvements are better than those achieved by other methods.

**Figure 7:**
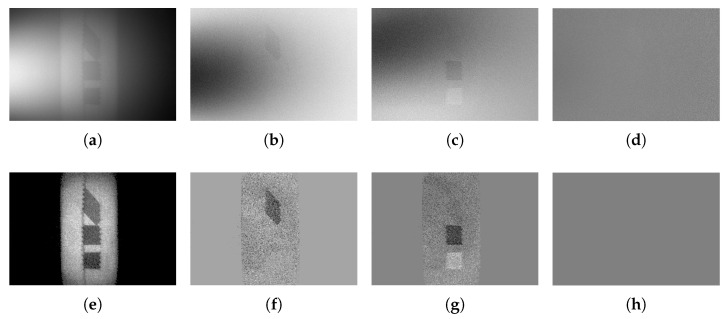
Polarization underwater imaging of three polarizers with different orientations. (**a**–**d**) are original underwater Stokes parameter images $\left[I_{total}\;Q_{total}\;U_{total}\;V_{total}\right]$. (**e**–**h**) are Stokes parameter images reconstructed by the proposed method $\left[I_{target}\;Q_{target}\;U_{target}\;V_{target}\right]$.

Traditional underwater polarization imaging methods can only provide clear DOP and intensity images of the ambient light and the object but cannot provide the distribution of the polarization state of the object and the ambient light [23]. However, in real underwater scenes, due to the complexity (anisotropy) of the object’s nature, the information of the polarization state (e.g., the directions of linearly polarized light and the circularly polarized component) rather than the DOP is more important and can provide more information. The method in this paper can reconstruct not only the light intensity and DOP images of the target but also the full polarization state image of the object, which can be visualized in the color-space by using the color-encoding method. To demonstrate that the proposed method can also provide clear images of the polarization state information of the objects underwater, three polarizers with different orientations were imaged by the proposed system and reconstructed by the proposed method, as shown in Figure 7. Three polarizers were placed on a tape as shown in Figure 7, where, from top to bottom, the polarization directions are manually set at around 90 degrees, 45 degrees, and 135 degrees, respectively. Figure 7a–d shows the raw intensity, Q, U, and V images of the sample, respectively. It can be seen that the three targets cannot be visualized clearly due to the background scattered light. By applying the proposed method to each Stokes channel (i.e., I, Q, U, and V), the target information can be extracted clearly in the Stokes parameters channels, as shown in Figure 7e–h. By removing the background light from each channel, the Q, U, V parameters of light scattered back from the polarizers from top to bottom are [−0.66 −0.13 0] (corresponding to 95.57 degrees of linearly polarized light), [0.12 −0.61 0] (corresponding to 129.44 degrees of linearly polarized light), and [0.04 0.55 0] (corresponding to 42.92 degrees of linearly polarized light), respectively, which are close to the practical values we manually set (i.e., 90 degrees, 135 degrees, and 45 degrees, respectively), demonstrating that this method can recover the full polarization state information of the target. It should be noted that due to the turbid water, the DOP values of the light scattered back from the three polarizers are decreased. However, it is hard to visualize the three targets simultaneously in a single Stokes parameters channel, as shown in Figure 7e–h.

**Figure 8:**
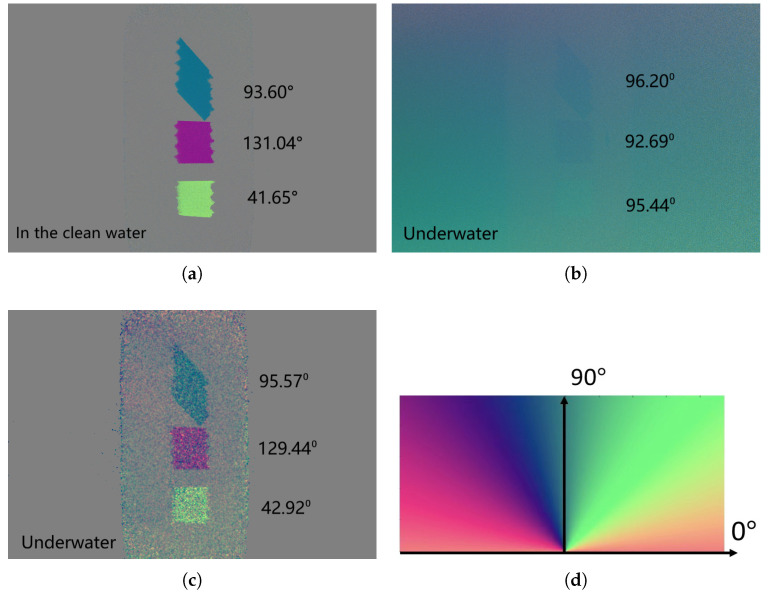
Color-encoded polarization state image of the 3 polarizers with different orientations. (**a**) Original polarization state of the sample in the clean water. (**b**) Original underwater polarization state image of the sample. (**c**) Clear color-encoded polarization state image of the sample reconstructed by the proposed method. (**d**) Colormap for the linearly polarized light.

To present the three targets with full polarization state information in one figure, the clear Q, U, and V images are encoded by the red, green, and blue, respectively, using Equation (12), as shown in Figure 8. Figure 8a is the polarization state image of the sample in the clean water. Without the optical distortion introduced by the turbid liquid, the three polarizers can be clearly seen. The Q, U, and V values of the three targets in the clean water from top to bottom are [−0.87 −0.11 0.10] (corresponding to 93.60 degrees of the linearly polarized component), [0.11 −0.79 0.10] (corresponding to 131.04 degrees of the linearly polarized component), and [0.08 0.68 −0.10] (corresponding to 41.65 degrees of the linearly polarized component), respectively. Figure 8b shows the polarization state of underwater images without any signal processing. The Q, U, and V values of the three targets from top to the bottom are [−0.5 −0.11 0.05] (corresponding to 96.20 degrees of the linearly polarized component), [−0.53 −0.05 0.05] (corresponding to 92.69 degrees of the linearly polarized component), and [−0.52 0.10 −0.05] (corresponding to 95.44 degrees of the linearly polarized component), respectively (Figure 8b), which is close to the polarization state of the ambient light. It can be seen that without the proposed method to remove the ambient light, the polarization states of the three objects cannot be separated from the ambient light. By applying the proposed method to obtain the intensity image, although the targets can be clearly seen in Figure 7e, the orientations of these three polarizers cannot be distinguished. In the clear color-encoded polarization state images, as shown in Figure 8c, the three polarizers with different orientations can be clearly distinguished by different colors. The colors and the quantitative orientations (mentioned in the above paragraph) of the three recovered targets (Figure 8c) are close to that of the three targets in the clean water (Figure 8a), demonstrating that the proposed method can effectively recover the polarization states of the objects. Moreover, according to the colormap in Figure 8d, the orientations of the polarization states scattered back from these three polarizers can be recognized intuitively and quantitatively in the color-encoded polarization state image. These results demonstrate that the proposed method can provide a clear underwater image of the target with full polarization state information effectively and quantitatively.

**Figure 9:**
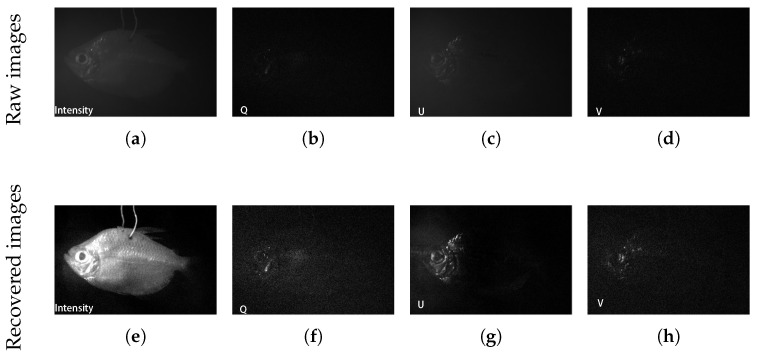
Underwater images of the fish in the turbid water with milk concentration of 8 mL captured by the full Stokes camera. (**a**–**d**) are the raw Stokes images of the fish $\left[I_{total}\;Q_{total}\;U_{total}\;V_{total}\right]$. (**e**–**h**) are the recovered clear images of the fish reconstructed by the proposed method $\left[I_{target}\;Q_{target}\;U_{target}\;V_{target}\right]$.

**Figure 10:**
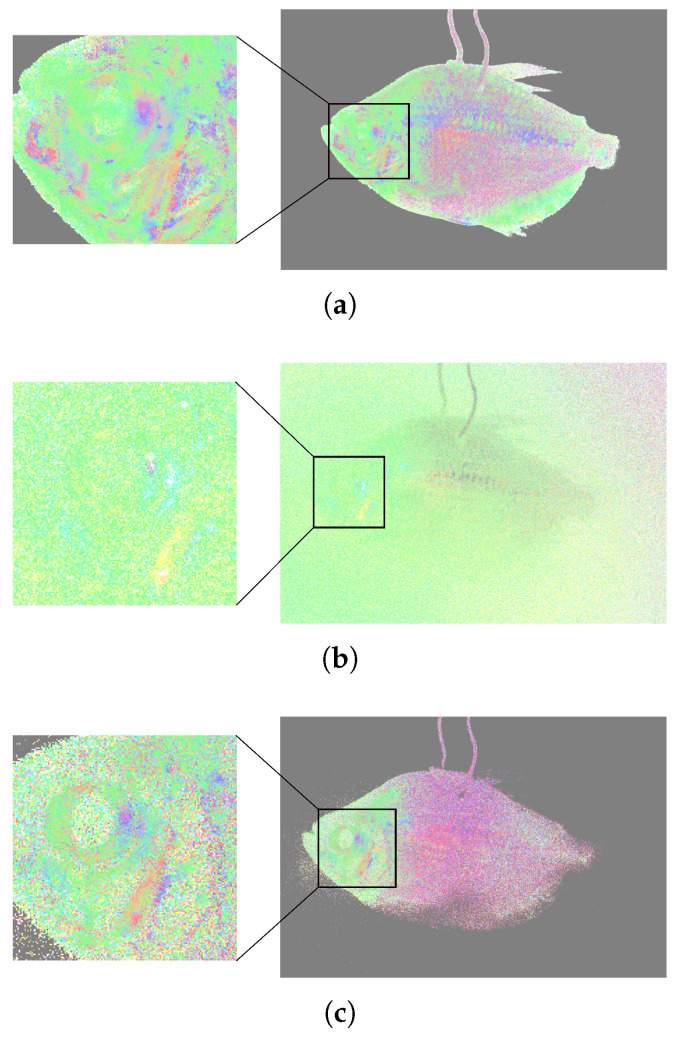
Color-encoded polarization state image of the fish. (**a**) Original polarization state of the sample in the clean water. (**b**) Original underwater polarization state image of the sample. (**c**) Clear color-encoded polarization state image of the sample reconstructed by the proposed method.

To further verify the versatility and applicability of our method, we imaged a fish (kissing gourami, adult, female) in clean and turbid water by using the proposed. Figure 9a–d shows the raw intensity, Q-component, U-component, and V-component image of the fish in the turbid water with a milk concentration of 8 mL. It is hard to differentiate the contour of the fish in the turbid water due to the strong scattering of the ambient light. Figure 9a–d shows the intensity, Q-component, U-component, and V-component obtained by the proposed method. It can be seen that the clear-intensity images of the fish can be separated effectively from the ambient light, as shown in Figure 9e. The polarization information, including the Q, U, and V components, can also be extracted from the ambient light. It should be noted that even when light is linearly polarized light when the sample is birefringent, the V-component cannot be neglected, as shown in Figure 9d,h. To further demonstrate that our method can effectively extract the polarization information of the sample, we utilized the color-encoded method to visualize the polarization state images of the fish in Figure 10. Figure 10a is the polarization state image of the fish in the clean water. Different colors show that the head of the fish is birefringent. Figure 10b shows the raw polarization state image of the fish in the turbid water. Compared with the polarization state image of the fish in the clean water, the polarization state information of the fish in the turbid water is totally disturbed by the ambient light, making it hard to discern the target’s polarization characteristics. Figure 10c shows the polarization state image of the fish in the turbid water using the proposed method, where it can be seen that the polarization state information of the fish can be recovered. These results show that by applying the proposed method, the polarization information of the fish was recovered, allowing the polarization state information of the sample to be better presented.

## 4. Discussion

Although the method proposed in this paper can provide a clear polarization state image in the underwater environment, there are still some issues that need to be addressed in future work. The method in this paper requires multiple shots, which may sacrifice the imaging time. Hence, the reduction of the number of shots under the premise of guaranteeing the separation effect is an area that can be improved in the future. Moreover, using a full Stokes camera with higher imaging speed can also mitigate this issue. The second issue is that the objects need to be sparse in the whole FOV. When the objects are too large in the whole FOV, the sparsity of the objects cannot be ensured, and hence, the separation of the ambient light may be incomplete or incorrect. In the experiment, we moved the light source camera system manually to acquire multiple images for this study. In the future, we can improve the automation level and image acquisition speed by installing a mobile platform to move the light source camera system.

## 5. Conclusions

In this paper, a facile method that can extract the clear object information from the ambient light in four Stokes parameters channels (I, Q, U, and V), respectively, and provide the clear polarization state image in the turbid water is proposed. This method is implemented by capturing multiple images of the objects from different angles by moving a full Stokes camera. Then, each Stokes parameters channel has multiple images from different imaging angles, which are utilized to construct a new low-rank and sparse matrix. Then, the ambient light and the object in each Stokes parameters channel can be separated as the low-rank part and the sparse part based on the ADMM method because of the correlation of the ambient light and the sparsity of the object in multiple images. Finally, the clear polarization state image of the object can be obtained in the color-space by using the color-encoding method [17]. The experimental results show that by considering and separating the polarization distribution of the ambient light and the object in the four Stokes parameters channels, respectively, this method can image the sample regardless of the DOP of the sample and provides a better imaging performance compared with the traditional methods. Most importantly, this method can provide a clear image with full polarization state information, including the circularly polarized component. We believe this method extends the applicability of polarization underwater imaging techniques in ocean exploration. 

## Figures and Tables

**Table 1:** Image evaluation parameter calculation of Figure 6.

	Raw	Treibit $\left(P_{S}=0\right)$	Treibit $\left(P_{S}=1\right)$	CLAHE	ICA	Ours
PSNR	8.05	10.37	11.14	7.52	14.47	17.46
EME	6.09	8.50	8.96	12.03	16.22	20.43
SSIM	0.4346	0.6035	0.5052	0.3781	0.5148	0.7156

## Data Availability

Data are contained within the article.
